# High Prevalence of *Tropheryma whipplei* in Lao Kindergarten Children

**DOI:** 10.1371/journal.pntd.0003538

**Published:** 2015-02-20

**Authors:** Alpha Kabinet Keita, Audrey Dubot-Pérès, Koukeo Phommasone, Bountoy Sibounheuang, Manivanh Vongsouvath, Mayfong Mayxay, Didier Raoult, Paul N. Newton, Florence Fenollar

**Affiliations:** 1 Aix Marseille Université, URMITE, UM63, CNRS 7278, IRD 198, Inserm 1095, Marseille, France; 2 UMR_D 190, Aix Marseille Univ-IRD-EHESP, Medical University, Marseille, France; 3 Lao-Oxford-Mahosot Hospital-Wellcome Trust Research Unit (LOMWRU), Microbiology Laboratory, Mahosot Hospital, Vientiane, Lao PDR; 4 Centre for Tropical Medicine, Nuffield Department of Clinical Medicine, Churchill Hospital, University of Oxford, Oxford, United Kingdom; University of Tennessee, UNITED STATES

## Abstract

**Background:**

*Tropheryma whipplei* is a bacterium commonly found in feces of young children in Africa, but with no data from Asia. We estimated the prevalence of *T. whipplei* carriage in feces of children in Lao PDR (Laos).

**Methods/Principal Findings:**

Using specific quantitative real-time PCR, followed by genotyping for each positive specimen, we estimated the prevalence of *T. whipplei* in 113 feces from 106 children in Vientiane, the Lao PDR (Laos). *T. whipplei* was detected in 48% (51/106) of children. Those aged ≤4 years were significantly less frequently positive (17/52, 33%) than older children (34/54, 63%; p< 0.001). Positive samples were genotyped. Eight genotypes were detected including 7 specific to Laos. Genotype 2, previously detected in Europe, was circulating (21% of positive children) in 2 kindergartens (Chompet and Akad). Genotypes 136 and 138 were specific to Chompet (21% and 15.8%, respectively) whereas genotype 139 was specific to Akad (10.55%).

**Conclusions/Significance:**

*T. whipplei* is a widely distributed bacterium, highly prevalent in feces of healthy children in Laos. Further research is needed to identify the public health significance of this finding.

## Author Summary

*Tropheryma whipplei* is a common bacterium carried in feces of young children. Here, using specific PCR, we estimated the prevalence of *T*. *whipplei* in 113 feces from 106 children in Vientiane, the Lao PDR (Laos). *T*. *whipplei* was detected in 48% (51/106) of children. Eight genotypes were detected, including 7 specific to Laos. Genotype 2, previously detected in Europe, was circulating (21% of positive children) in 2 kindergartens (Chompet and Akad). Genotypes 136 and 138 were specific to Chompet (21% and 15.8%, respectively), whereas genotype 139 was specific to Akad (10.55%). Long regarded as a rare bacterium, now we can affirm that *T*. *whipplei* is a widely distributed bacterium, highly prevalent in feces including those from children in Vientiane.

## Introduction

*Tropheryma whipplei* is a common bacterium found in human feces; its prevalence depends mainly on age, exposure to saliva and/or human feces, and geographical area [1–8]. The prevalence in feces is estimated to be between 1 and 11% of European adults, but reaches 75% in children <4 years old in rural Senegal and 38% in relatives of those with Whipple’s disease or chronic carriers of the bacterium in France [1,3,5,7]. *T*. *whipplei* carriage was estimated to be 12.9% in homeless people in France and between 12 to 26% among European sewerage workers [1,6,9]. In France, this bacterium was also detected in the feces of 15% of children aged 2 to 4 years with gastroenteritis but not in control subjects of the same age [8].

*T*. *whipplei* is viable in culture on Specific Axenic Medium (SAM) using the feces of patients with Whipple's disease [10] and data strongly suggest that it is contagious [3,5,6,8,11,12]. Indeed, specific clones were observed with a high prevalence in children from the same city with gastroenteritis, among individuals from the same village in Senegal, among relatives of patients with Whipple’s disease and chronic carriers and among homeless people living in the same temporary visitor centre [3,5,6,8,11].

Recent studies suggest that *T*. *whipplei* infection is probably contracted during childhood [12]. There is a high prevalence of *T*. *whipplei* in rural Senegalese children <4 years old, up to 75%, that decreases to 30% among the 5–10 years old [12]. Moreover, in Senegal for the majority of children <6 years old, but not for those >6 years old, the presence of an immune response against the bacterium is consistently associated with the presence of the bacterium in their feces. This suggests that primary infection occurs before 5 years of age.

*T*. *whipplei* is widespread and many individuals are exposed to this bacterium in Africa and Europe [1–3,5–9,11–14]; almost 72% of individuals in Senegal and 50% in France carried antibodies against the bacterium [3,5]. *T*. *whipplei* is involved in multifaceted conditions, including acute and chronic infections [15]. Although many are exposed to *T*. *whipplei*, few develop disease, possibly due to as yet undelineated predisposing immuno-genetic factors [2,3,5–7,11,12,15,16].

Data about *T*. *whipplei* in Asia are few, although *T*. *whipplei* has been reported in the saliva of healthy Koreans [17–20]. We therefore sought to determine the prevalence of *T*. *whipplei* carriage and identify genotypes circulating among children in Laos.

## Materials and Methods

### Population recruitment and ethical statement

**Fig 1:**
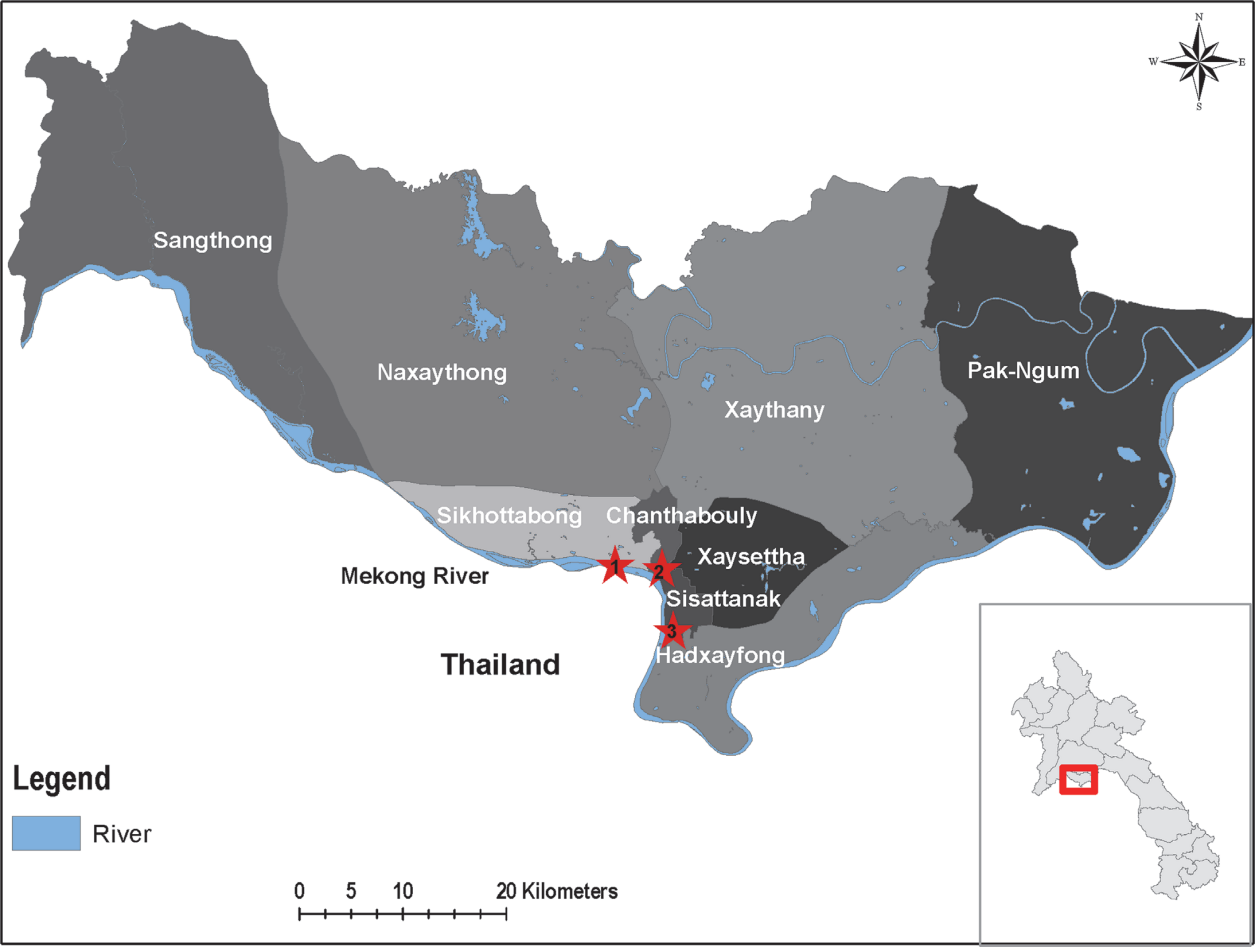
A map showing the geographical localization of the different study sites. The red stars symbolize the study sites. 1: Sailoum, 2: Chompet, 3: Akad.

The initial objective of this study was to look for enterovirus carriage in Lao kindergarten children. It is only secondarily that the carriage of *T*. *whipplei* has been investigated in children. For this purpose, we analyzed the feces from healthy children collected between 2010 and 2012 in the framework of enterovirus study [21] in 3 kindergartens in Vientiane City, Lao PDR (Laos), located in 3 different villages (Sailom, Chompet, and Akad) (Fig. 1). Although children were present at the kindergarten the day of sampling and thus considered healthy, they were also examined for conjunctival suffusion, pharyngeal erythema, rhinitis or runny nose, diarrhea, vomiting, mouth ulcer, vesicle on hands, and vesicles on feet.

Fresh feces were collected in sterile containers from children aged 1–7 years whose guardians/parents gave informed written consent. After collection, each specimen was stored at+4°C up to 24 hours. Each specimen was vortexed in MEM medium (Minimum Essential Medium, Invitrogen, Carlsbad, CA, USA), that is a commonly used in Lao laboratory as viral transport medium as recommended by WHO, with glass beads for 1 minute. Then, after 30 minutes centrifugation at 3,000g at +4°C (adaptation of the protocol from the polio laboratory manual 2004 WHO/IVB/04.10 with the replacement of chloroform by antibiotics and fungizone), the supernatant was recovered and stored at -80°C for culture of enterovirus but the presence of antibiotics in the transport medium prevented the culture of *T*. *whipplei*. After enterovirus screening in Laos, 113 feces, from 106 children, were shipped on dry ice to virology laboratory in Marseille, France, for enterovirus confirmation (94/113, 83% were confirmed to be positive for enterovirus), all were positive for β-actin and included in the present study.

The study was granted ethical approval by the Lao National Ethics Committee for Health Research (331/NECHR) and by the Oxford Tropical Research Ethics Committee (OXTREC 020–10), with amendments from both committees for feces collection for *T*. *whipplei*.

### Molecular assays

DNA was extracted from 100μl of each sample using Machery Nagel DNA Mini Kit (Machery Nagel Gmbh & Co., Düren, Germany) in accordance with the manufacturer’s instructions. Quantitative real-time PCR (qPCR) was performed using CFX96TM Real-Time PCR Detection System (Bio-Rad Laboratories, Hercules, CA, USA) with the FAST qPCR Master mix No ROX (Eurogentec, Liege Science Park, Belgium).

DNA extraction quality was verified for all specimens by qPCR targeting a gene encoding β-actin using primer pair ActinF (5’-CATGCCATCCTGCATCTGGA-3’) and ActinR (5’-CCGTGGCCATCTCTTGCTCG-3’) associated with a specific TaqMan probe (6-FAM-CGGGAAATCGTGCGTGACATTAAG-TAMRA). Negative β-actin specimens were excluded from analysis as previously described [22,23].

For *T*. *whipplei* detection, specimens were first tested using qPCR targeting a repeated sequence with the Twhi3F (5'-TTGTGTATTTGGTATTAGATGAAACAG-3’) and Twhi3R (5'-CCCTACAATATGAAACAGCCTTTG-3’) primer pair and the specific TaqMan probe Twhi3 (6-FAM-GGGATAGAGCAGGAGGTGTCTGTCTGG-TAMRA). When a sample was positive with this assay, the result was confirmed using a second qPCR targeting another repeated sequence with the Twhi2F (5'-TGAGGATGTATCTGTGTATGGGACA-3’) and Twhi2R (5'-TCCTGTTACAAGCAGTACAAAACAAA-3’) primer set and the Twhi2 probe (6-FAM-GAGAGATGGGGTGCAGGACAGGG-TAMRA). To validate each assay, qPCR mix was systematically used as a negative control and *T*. *whipplei* DNA as positive control, as previously reported [24,25].

Genotyping of *T*. *whipplei* was attempted for each positive specimen and performed as described [26]. Briefly, four highly variable genomic sequences (HVGSs) of *T*. *whipplei* were amplified and sequenced. The genotype was obtained after the analysis of each four HVGSs obtained from each specimen and compared with those available in both the GenBank database and our internal laboratory database in order to determine their corresponding genotype.

### Statistical analysis

PASW statistics 17 software (SPSS, Chicago, IL, USA) was used for data analysis and non-parametric values were compared using χ2 or the Fisher’s exact tests. Statistical significance was defined as *p*<0.05.

## Results

We analyzed 113 feces from 106 children (59 [56%] females) aged 1–7 years (mean 4 standard deviation [SD] ± 1.22 years). Fifty three of 106 children (50%) were from Chompet, 35 (33%) from Akad, and 18 (17%) from Sailom kindergartens. Among 106 children, 51 (48%, 95% CI 39–58) had a positive qPCR for *T*. *whipplei*. The prevalence of positive samples in Chompet was 27/53 (51%, 95% CI 38–64), that was not statistically different (p = 0.506, OR 1.2, 95% CI 0.58–2.57) to those from Akad and Sailom with 17/35 (49%, 95% CI 32–65) and 7/18 (39%, 95% CI 19–62) positive, respectively. Seven children provided more than one fecal sample; 3 were negative in the first year but positive the following year. Two were positive for 2 years and 2 were negative for 2 consecutive years.

**Fig 2:**
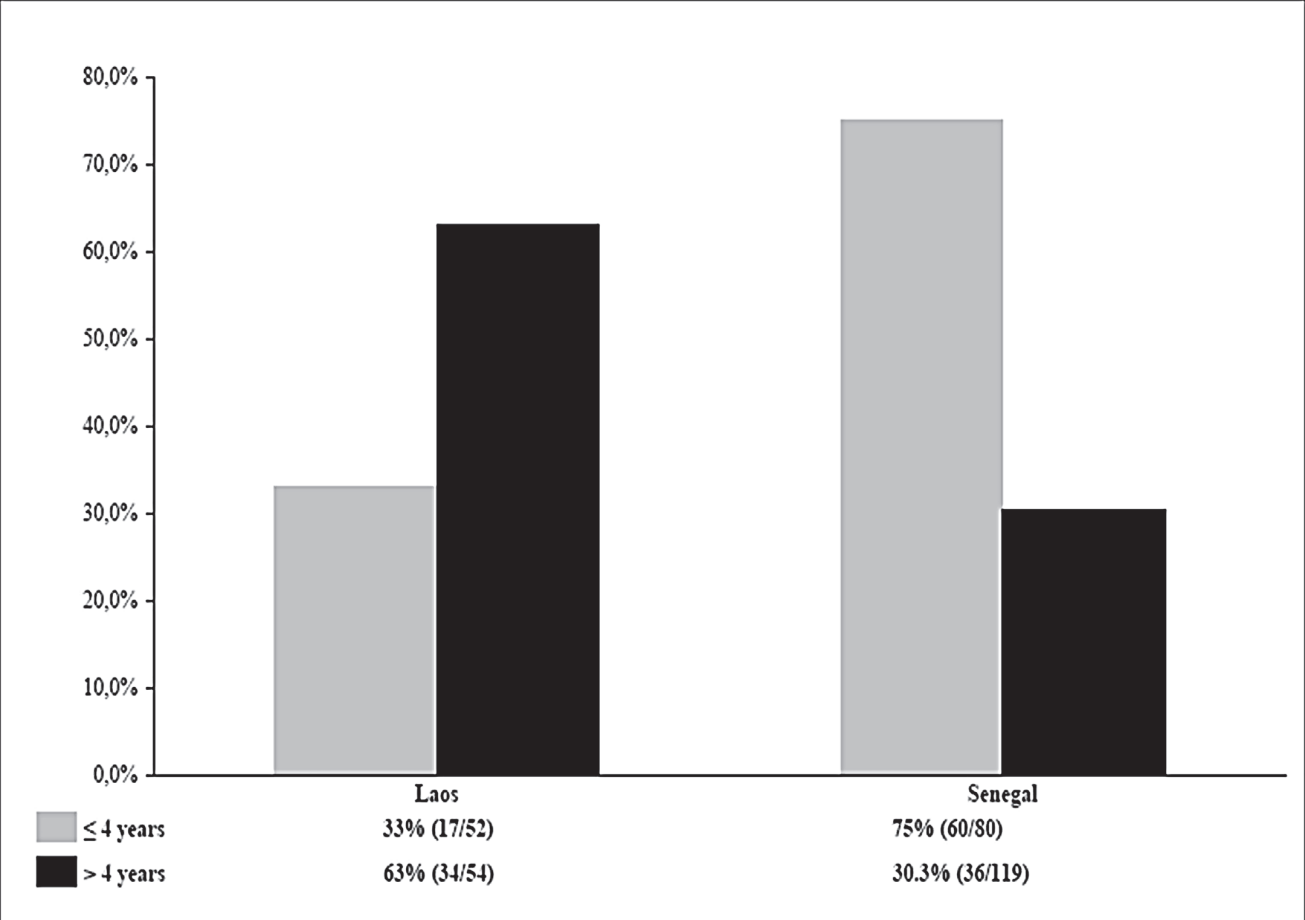
Comparisons of the prevalence of *T*. *whipplei* carriage in children feces between Senegal and Laos.

Children aged ≤4 years were significantly less frequently positive for *T*. *whipplei* (17/52, 33%) than older children (34/54, 63%; *p*<0.001, OR 0.12, 95% CI 0.29–0.68). The prevalence of *T*. *whipplei* carriage in feces from Lao children and those from rural Senegal are summarized in Fig. 2 [5]. Lao children aged ≤4 years were significantly less frequently positive for *T*. *whipplei* than Senegalese children (33% versus 75%, *p*<0.001, OR 0.16, 95% CI 0.07–0.37) whereas Senegalese children aged >4 years were significantly less frequently positive for *T*. *whipplei* than Senegalese children (30.3% versus 63%, *p*<0.001, OR 4.25, 95% CI 2.04–8.92).

**Fig 3:**
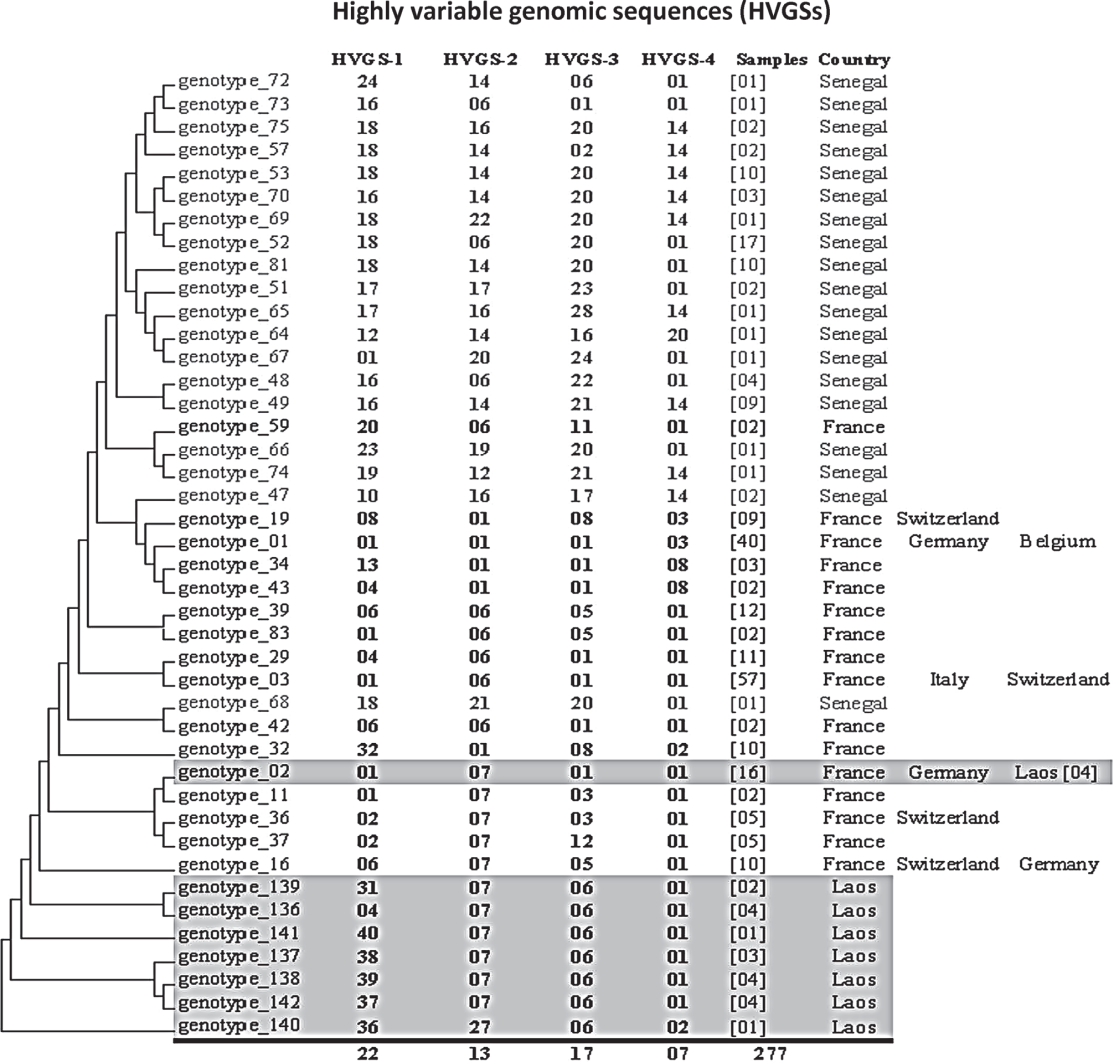
Genetic diversity of 42 genotypes of *Tropheryma whipplei* obtained from 277 samples. Phylogenetic trees were constructed using the maximum likelihood method based on the Tamura 3-parameter substitution model. The countries in which the genotype has been detected are presented opposite the genotype, and the number of different individuals in which the genotype has been detected is provided in parentheses. The genotypes of each highly variable genomic sequence (HVGS) have intervened in 22, 13, 17 and 7 different combinations, respectively.

Among 51 Lao children *T*. *whipplei* positive, genotypes were obtained for 19 samples. We obtained 8 different genotypes. Genotype 2 has been detected in France and Germany but 7 were new genotypes described from Laos for the first time (genotypes 136, 137, 138, 139, 140, 141, and 142; Fig. 3). Genotype 2 was detected in 4/19 (21%) children, 3 were from Chompet and one from Akad kindergarten. New genotypes 136 and 138 were both detected in Chompet kindergarten in 4/19 (21%) and 3/19 (15.8%), respectively, whilst genotype 139 was identified in Akad kindergarten in 2/19 (10.5%). The new genotypes 140, 141, and 142 were each detected in one child in each kindergarten (Fig. 3). Only one genotype (137) occurred in all three kindergartens, but was only identified once in each. From the two children positive on two different occasions, we obtained successive genotypes from only one child; in whom the same genotype (138) was found after an 8 month interval.

Among the children included in this study, only 1 presented pharyngeal erythema and 15 rhinitis. All the other symptoms, including diarrhea, were not observed in any of the children. Among the 51 children positive for *T*. *whipplei*, 5 exhibited rhinitis (9.8%).

## Discussion

We describe *T*. *whipplei* in the stools of apparently healthy children for the first time in Asia. The data are based on strict experimental procedures, including positive and negative controls to validate each assay. The positivity of the β-actin gene was checked and confirmed for each included sample [2] and each *T*. *whipplei* qPCR was systematically confirmed by the successful amplification of another specific DNA sequence. Overall, the prevalence of *T*. *whipplei* carriage in feces from Lao children aged <7 years is 48% [5]. The only symptom observed among children positive for *T*. *whipplei* was rhinitis (9.8%) but this symptom was also observed in 16.1% of the negative children. Thus, no link between the presence of rhinitis and that of the bacterium can be established.

Lao children were contaminated with *T*. *whipplei* significantly later than those of rural Senegal. In France *T*. *whipplei* DNA was not detected in feces of healthy children <6 years old [8]. As far as we aware no one has investigated *T*. *whipplei* fecal carriage in Asia, but it has been detected in Korea among 1.5% of healthy peoples’ saliva, but not in any of 108 small-bowel biopsies in Malaysia or in any of ten marginal supragingival plaque samples from Chinese patients with gingivitis [17,18,20]. In rural Senegal, the first contact with the bacterium occurred mainly in children before 4 years of age, with the prevalence of *T*. *whipplei* in feces declining from 75% in those ≤4 years to 30.3% among those >4 years [5]. This trend was confirmed by a recent study performed in Gabon, with a high prevalence of *T*. *whipplei* in children <5 years old, up to 40%, with a lower prevalence (36.4%) among those aged 5–10 years and 12.6% among those of 11–20 years [27]. In contrast, in Laos we observed a significantly lower prevalence in children ≤4 years (33%) than in children >4 years (63%).

A current hypothesis is that *T*. *whipplei* is transmitted between humans according to hygiene conditions [5,7], through saliva (oral-oral transmission) if hygiene conditions are good, or feces (fecal-oral transmission) if hygiene poor [28]. *T*. *whipplei* has rarely been identified in environmental samples and humans appear to be the main reservoir and source of this bacterium in rural Senegal [7,12]. Poor sanitary conditions and close contacts between children from the earliest age could explain the difference in exposure to *T*. *whipplei* between Senegalese and Gabonese children versus Laotian children for whom contacts with other children are probably much less common before entering kindergarten [7,27]. In Senegal, households with high prevalence of *T*. *whipplei* carriage had also significantly less access to toilets, supporting the inter-human transmission of the bacterium being from human feces through hand transmission. In Laos, toilets were present at home for all included children and also at kindergarten. However, no information was available about the hand washing practice. Finally, close contact could play a role in transmission of *T*. *whipplei* as well as the lack of hand hygiene [6].

In Europe and Africa, 135 different genotypes have been identified from 359 people positive for *T*. *whipplei*. Eight genotypes of *T*. *whipplei*, including 7 newly described, were identified in feces from healthy Lao children. Previous work showed that *T*. *whipplei* genotypes found in Senegal and in Europe were different [2,4–6]. Genotype 2 was detected in 4/19 (21%) Lao children, 3 were from Chompet and one from Akad kindergarten. This genotype had been previously detected in 12 out of 359 other people (3.34% versus 21%; *p*<0.001) in Europe, but not in Senegal. The presence of highly prevalent clones specific to some kindergartens, such as genotypes 136 and 138 in Chompet kindergarten, suggest that these clones are probably epidemic and support the contagiousness of *T*. *whipplei*. Furthermore, it is important to underline that currently no specific genotypes have been associated with disease versus asymptomatic carriage. Besides, a same genotype may be observed in acute infections, chronic infections and/or asymptomatic carriage [4]. Overall, *T*. *whipplei* genotyping is a useful tool to differentiate a relapse of Whipple’s disease to a reinfection with another genotype but also to detect circulating or epidemic clones [3–6,8,26,29,30].

Chronic infections due to *T*. *whipplei* such as classic Whipple’s disease or endocarditis have been scarcely reported in Asia and no such patients have been reported from Laos or adjoining North East Thailand [31]. To the best of our knowledge, only two Japanese patients and three individuals from India with Whipple's disease have been reported in the literature [31,32]. These data reinforces that although many people are exposed to *T*. *whipplei*, only few individuals develop chronic infection [4]. The prevalence of *T*. *whipplei* carriage is high in areas where poor sanitary conditions and close contacts are observed but *T*. *whipplei* seems to be an opportunistic bacterium that causes chronic infections probably linked to an, as yet unknown, specific immunological defect. Indeed, several dysregulated T-cell functions, a persistent deficiency in, or the absence of, a T. whipplei-specific T-helper cell-1 response have been observed in the patients with classic Whipple’s disease [33]. Furthermore, the susceptibility of patients to Whipple’s disease, with relapses and reinfections with new strains, along with a lack of, or a weak, serological response supports also a specific T. whipplei immunodeficiency [30,34]. Several observations support a genetic predisposition for Whipple’s disease. Indeed, the majority of patients reported to date are Caucasian, mainly males [4,29,35,36]. It is also remarkable for a rare disease that six familial proven cases of Whipple’s disease have been reported in the literature [15,36,37]. A higher frequency of the HLA alleles DRB*13 and DQB1*06 in patients with classic Whipple’s disease has been observed [38]. All these data may explain why *T*. *whipplei* carriage is so high in Africa and Asia relative to Europe, whereas there are so few or no confirmed cases of chronic Whipple's disease in these populations.

The high prevalence of *T*. *whipplei* carriage in the feces of Lao children, confirms that this bacterium occurs in South East Asia and that infection occurs in childhood. Further research is needed to better understand the natural history, public health significance, and effect of *T*. *whipplei* on the health in Laos.

## Supporting Information

S1 Checklist STROBE Checklist. (DOC)
