# Supplementary material for: High Prevalence of Tropheryma whipplei in Lao Kindergarten Children
Source: PLoS Negl Trop Dis. 2015 Feb 20;9(2):e0003538. doi: 10.1371/journal.pntd.0003538 (PMC4336285; doi:10.1371/journal.pntd.0003538)
Supplement: S1 Checklist — (DOC) [file pntd.0003538.s001.doc]

**STROBE Statement—Checklist**

|  | Item No | Recommendation |
| --- | --- | --- |
| **Title and abstract** | 1 | (*a*) High prevalence of *Tropheryma whipplei* in Lao kindergarten children. |
|  |  | (*b*) An informative and balanced summary of what was done and what was found is provided in the abstract of our manuscript. |
| Introduction | | |
| Background/rationale | 2 | *Tropheryma whipplei* is a bacterium commonly found in feces of young children in Africa but data about this bacterium in Asia are few. |
| Objectives | 3 | Estimation of the prevalence of *T. whipplei* carriage and identification of *T. whipplei* genotypes circulating in feces of children in Lao PDR (Laos). |
| Methods | | |
| Study design | 4 | Feces from healthy children collected between 2010 and 2012 in the framework of enterovirus study in Lao PDR (Laos). |
| Setting | 5 | The study was located in 3 kindergartens in 3 different villages (Sailom, Chompet, and Akad) in Lao PDR. |
| Participants | 6 | The eligibility criteria were healthy children present in 3 kindergartens whose guardians/parents gave informed written consent |
| Variables | 7 | The presence of *T. whipplei* was detected using specific quantitative real-time PCR assays, followed by genotyping for each positive specimen. |
| Data sources/ measurement | 8* | Each specific quantitative real-time PCR assays was systematically confirmed using another specific assay. |
| Bias | 9 | The quality of DNA extracts could be a potential source of bias. The β-actin gene was checked for each included sample in the present study and all were positive confirming the DNA quality. |
| Study size | 10 | 113 feces from 106 children |
| Quantitative variables | 11 | Explain how quantitative variables were handled in the analyses. If applicable, describe which groupings were chosen and why |
| Statistical methods | 12 | PASW statistics 17 software (**SPSS, Chicago,** IL, USA) was used for data analysis and non-parametric values were compared using χ2 or the Fisher’s exact tests. Statistical significance was defined as *p*<0.05. |
| Results | | |
| Participants | 13* | We analyzed 113 feces from 106 healthy children from 3 kindergartens in Lao PDR. |
| Descriptive data | 14* | The 106 healthy children were aged from 1-7 years (mean 4 standard deviation [SD] ± 1.22 years); 59 [56%] were females. Fifty three of 106 children (50%) were from Chompet, 35 (33%) from Akad, and 18 (17%) from Sailom kindergartens. No data was lacking |
| Outcome data | 15* | Not applicable |
| Main results | 16 | *T. whipplei* was detected in 48% (51/106) of children. Those aged ≤4 years were significantly less frequently positive (17/52, 33%) than older children (34/54, 63%; *p*< 0.001). Positive samples were genotyped. |
| Other analyses | 17 | Among 51 Lao children *T. whipplei* positive, genotypes were obtained for 19 samples. Eight genotypes were detected including 7 specific to Laos. Genotype 2, previously detected in Europe, was circulating (21% of positive children) in 2 kindergartens (Chompet and Akad). Genotypes 136 and 138 were specific to Chompet (21% and 15.8%, respectively) whereas genotype 139 was specific to Akad (10.55%). |
| Discussion | | |
| Key results | 18 | We report, for the first time, that *T. whipplei* was highly prevalent in the stools of healthy children in Laos, Asia*.* |
| Limitations | 19 | Only 3 kindergartens in Lao PDR were investigated. |
| Interpretation | 20 | *T. whipplei* is a widely distributed bacterium, highly prevalent in feces of healthy children in Laos. |
| Generalisability | 21 | This work can be extended to a larger sample including both children and adults and associated with an environmental study to better understand the natural history, public health significance, and effect of *T. whipplei* on the health in Laos. |
| Other information | | |
| Funding | 22 | We thank the Agence National de Recherche grant 2010 (MALEMAF) and the Institut Hospitalo-Universitaire Méditerranée Infection for their financial support. The Lao-Oxford-Mahosot Hospital-Wellcome Trust Research Unit is supported by the Wellcome Trust of Great Britain. |

*Give information separately for exposed and unexposed groups.

**Note:** An Explanation and Elaboration article discusses each checklist item and gives methodological background and published examples of transparent reporting. The STROBE checklist is best used in conjunction with this article (freely available on the Web sites of PLoS Medicine at http://www.plosmedicine.org/, Annals of Internal Medicine at http://www.annals.org/, and Epidemiology at http://www.epidem.com/). Information on the STROBE Initiative is available at http://www.strobe-statement.org.
